# Ultrafast Control of Néel Vector in Collinear Antiferromagnet MnPt

**DOI:** 10.1002/advs.202519395

**Published:** 2025-11-10

**Authors:** Sambhu Jana, Sobhan Subhra Mishra, James Lourembam, Ranjan Singh

**Affiliations:** ^1^ Division of Physics and Applied Physics School of Physical and Mathematical Sciences Nanyang Technological University Singapore 637371 Singapore; ^2^ Centre for Disruptive Photonic Technologies The Photonics Institute Nanyang Technological University Singapore 639798 Singapore; ^3^ Institute of Materials Research and Engineering (IMRE) Agency for Science Technology and Research (A^*^STAR) 2 Fusionopolis Way Singapore 138364 Singapore; ^4^ Department of Electrical Engineering University of Notre Dame Notre Dame IN 46556 USA

**Keywords:** Antiferromagnets, Néel vector manipulation, Light induced torque, Ultrafast AFM Spintronics

## Abstract

Exchange‐bias‐coupled spintronic terahertz emitters (EBC‐STEs) utilize an antiferromagnet (AFM) as both a passive pinning layer for ferromagnetic spins and a detector of spin currents. Conventionally, terahertz (THz) emission from spintronic terahertz emitters (STEs) is attributed solely to the ferromagnetic subsystem, with the ultrafast magnetization dynamics of AFM considered undetectable due to its zero net magnetic moment. Here, we demonstrate a novel experimental approach to probe and control ultrafast magnetization dynamics driven by laser‐induced optical torque on the Néel vector in collinear AFM MnPt. The Néel vector induced transient magnetization is isolated from ferromagnetic magnetization dynamics by detecting the distinct THz emission from the canted magnetic moment, as confirmed in Pt/MnPt bilayers. We further investigate the stability of the EBC‐STE compared to conventional STEs under optical excitation. This work provides direct evidence of ultrafast AFM magnetization dynamics in an EBC‐STE, transforming it from a passive emitter into a sensitive probe of interfacial ultrafast magnetism and unlocking the potential of antiferromagnetic THz spintronics.

## Graphical abstract

Direct evidence of ultrafast manipulation of the antiferromagnetic order parameter (Néel vector) in an exchange bias system is demonstrated using terahertz (THz) emission spectroscopy. Laser‐induced torque cants the Néel vector in the collinear antiferromagnet MnPt, generating a transient magnetic moment that produces THz radiation. This study provides an efficient mechanism for controlling antiferromagnetic spins with light.

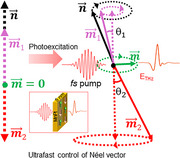

## 1 Introduction

The development of an EBC‐STE marks a significant advancement for miniaturized, high‐efficiency, and broadband THz sources, such as on‐chip THz sources and modulators.^[^ ^1^, ^2^ ^]^ This heterostructure, typically consisting of an antiferromagnet (AFM) and a ferromagnet (FM)^[^ ^3^, ^4^ ^]^ leverages the unidirectional exchange bias from the AFM to pin the magnetization direction of the FM layer. This exchange‐induced pinning eliminates the requirement of an external magnetic field to saturate the magnetization of the FM, a significant drawback of conventional FM/heavy metal (HM) emitters.^[^ ^5^, ^6^, ^7^ ^]^ In this established paradigm, the role of the AFM is purely passive: it is considered a static source of spin detector due to its strong spin‐orbit coupling (SOC) strength^[^ ^8^ ^]^ or simply a means to bias the FM layer magnetically.^[^ ^9^ ^]^ The resulting THz emission is universally attributed to ultrafast spin‐to‐charge conversion within the AFM/FM system, with the magnetization of AFM dynamics deemed negligible and undetectable due to its zero net magnetic moment.^[^ ^10^ ^]^

However, it overlooks a few critical scientific questions. First, the true role of the AFM layer during ultrafast photoexcitation. Second, the robustness of the magnetic moment and interfacial equilibrium in the exchange bias‐coupled system under femtosecond laser excitation. In previous studies, the dynamics of this AFM order parameter (Néel vector) in the THz emission process have been entirely overlooked.^[^ ^1^ ^]^ However, the investigation of the ultrafast magnetic behavior of AFM is critical due to its high intrinsic resonance frequency^[^ ^11^ ^]^ and minimal stray field.^[^ ^12^ ^]^ The key to harnessing the AFM spin dynamics lies in the effective control and, importantly, the detection and manipulation of the Néel vector. While femtosecond laser excitation can induce ultrafast effects in AFMs, reading out these changes is hard because AFMs have no net magnetization, making direct measurement challenging. Techniques like neutron diffraction^[^ ^13^ ^]^ and time‐resolved XMCD (X‐ray magnetic circular dichroism) are commonly used for probing magnetic structures and dynamics in AFMs.^[^ ^14^, ^15^ ^]^ However, these methods often require large‐scale synchrotron facilities for sensitivity and specificity. Recently, ultrafast magnetization dynamics in non‐collinear and symmetry‐broken AFMs have been explored through THz emission, where the underlying mechanism is attributed to the inverse spin Hall effect (ISHE)^[^ ^16^, ^17^ ^]^ or inverse Rashba Edelstein effect.^[^ ^18^ ^]^

In this work, we propose an alternative to this established paradigm. We present for the first time direct evidence of ultrafast light‐induced Néel vector dynamics in a collinear AFM (MnPt) and demonstrate its active contribution to THz emission within an EBC‐STE (MnPt/CoFeB). We propose a novel experimental design that isolates the weak, transient magnetic moment of the photoexcited AFM layer. The ultrafast laser pulses exert a torque directly on the AFM Néel vector, driving its dynamics, which are then transduced into a THz signal. This theoretical hypothesis of the THz emission mechanism is proven by experimental results. Finally, we demonstrate the exceptional robustness of the exchange bias system under intense ultrafast laser perturbation. Our experiments reveal that AFM is not a passive element but plays an active role in the THz generation process. Hence, an exchange bias system is critical for detecting laser‐induced transient magnetization in antiferromagnets, enabling the study of ultrafast spin dynamics and Néel vector control, and advancing understanding of field‐free terahertz spintronic mechanisms despite minimal emission signals.

## 2 Results and Discussion

**Figure 1:**
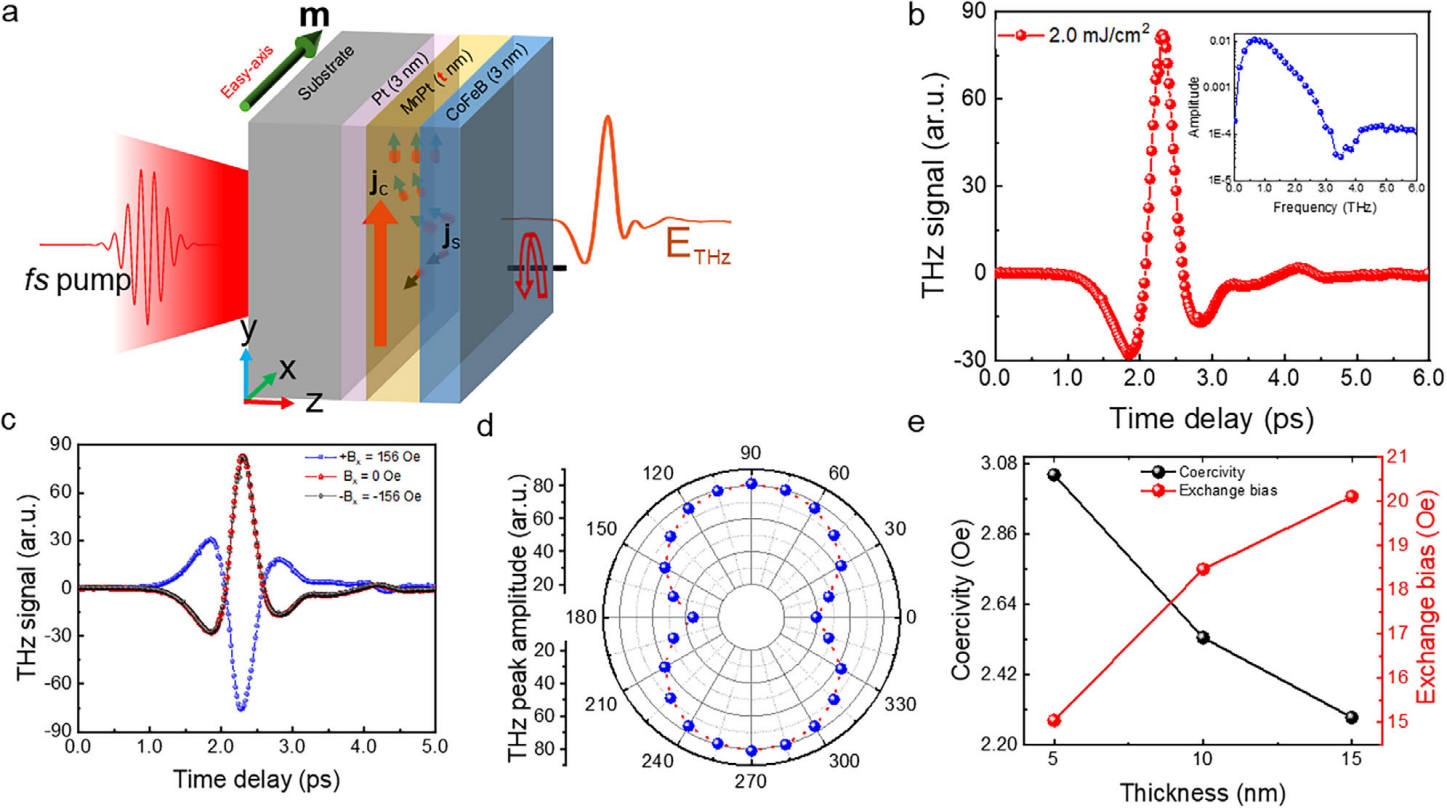
Significance of exchange bias in THz emission from an EBC‐STE composed of Pt/MnPt/CoFeB. a) Schematic portrayal of THz emission from an AFM‐FM heterostructure, with an 800 nm fs laser pulse incident normally on one side of the sample, leading to THz pulse emission from the opposite side. b) The THz time signal in the Pt (3 nm)/MnPt (5 nm)/CoFeB (3 nm) sample demonstrates strong broadband emission, with the corresponding fast Fourier transform (FFT) shown in the inset. c) The magnetic field‐dependent THz shows that the device functions as a truly field‐free emitter, as the emission amplitude remains unchanged under an applied field. However, the difference in THz amplitude between positive and negative fields indicates strong pinning of the ferromagnetic moment at the interfaces along the easy‐axis direction. Consequently, when the field is reversed from the easy axis, the magnetic domains cannot fully realign with the new field direction. d) The C2‐symmetric rotation of the THz peak‐to‐peak amplitude reflects unidirectional magnetic anisotropy, a clear signature of exchange bias. e) Increasing the antiferromagnetic layer thickness enhances the exchange bias field through stronger pinning of ferromagnetic spins along the AFM anisotropy axis, enabled by strengthened FM‐AFM interfacial exchange interactions. This enhanced bias lowers the energy barrier for ferromagnetic domain reversal, thereby causing a gradual decrease in coercivity.

THz emission spectroscopy is used to study magnetization dynamics of an EBC‐STE sample made of Pt (3 nm)/MnPt (t nm)/CoFeB (3 nm), as illustrated in **Figure** **1**a. In this heterostructure, the thin Pt film acts as a buffer layer, providing a smooth, lattice‐matched surface that helps MnPt form strong antiferromagnetic order during growth.^[^ ^19^ ^]^ The sample is grown under an in‐plane field to set a preferred magnetic orientation. The primary mechanism for THz emission in this EBC‐STE is inverse spin hall effect (ISHE), similar to what occurs in FM/HM heterostructures.^[^ ^20^ ^]^ An ultrafast laser pulse excites the heterostructure, generating spin‐polarized hot electrons in the CoFeB layer. These spin‐polarized hot electrons, i.e., spin currents (***j***_s_), are injected into the adjacent MnPt layer via the superdiffusive transport.^[^ ^21^ ^]^ MnPt, which is an efficient spin detector because of its strong SOC,^[^ ^8^ ^]^ converts these spin currents into charge currents through the ISHE effect. The resulting transient transverse charge currents (***j***_c_) are directed perpendicular to the magnetization (***m***) direction of the ferromagnetic layer. The spin‐to‐charge conversion process occurs entirely within the MnPt layer, since the spin diffusion length of MnPt is ≈0.5 nm,^[^ ^22^ ^]^ and no spin current reaches the adjacent Pt layer. Thus, the Pt layer does not directly influence the THz emission process but only supports the growth of MnPt.

Figure 1b and its inset show the THz time‐domain signal and corresponding frequency spectrum, respectively, for a Sub/Pt (3 nm)/MnPt ( nm)/CoFeB (3 nm) sample at a fluence of ≈2.0 mJ cm^−^^2^, without any external magnetic field. The measurement is performed with different magnetic field conditions to study the significance of exchange bias in the terahertz emission process. In Figure 1c, the THz peak amplitude remains identical with and without an external negative magnetic field. When the external magnetic field is reversed, the time‐domain THz signal undergoes a phase flip as expected, confirming that the emitted THz radiation is of magnetic origin. However, the peak amplitude is reduced by ≈7% compared to the signals observed in the negative field or field‐free conditions. This amplitude reduction in one magnetic field direction compared to the other strongly indicates the influence of an exchange bias field in the emitter. Specifically, under a positive external magnetic field, the magnetic dipoles of the FM layer align opposite to the direction of the uniaxial anisotropy. However, the exchange interaction between the AFM and FM spins at the interface competes with the ferromagnetic spin alignment along its preferred axis, i.e., the easy axis.^[^ ^1^, ^23^ ^]^ This interplay between the external bias field and the internal exchange bias weakens the overall THz signal, reducing the THz peak amplitude.

The azimuthal dependence of THz emission is also checked in the absence of an external magnetic field, as shown in Figure 1d. The results show a distinct *C_2_*‐symmetry, obtained by rotating the sample in the *xy*‐plane from 0° to 360° . The direction and polarity of the emitted THz field are directly determined by the direction of the pinned magnetization set by the exchange bias. Unlike in an isotropic system, where the emission is symmetric with respect to magnetization reversal, the exchange bias introduces a preferred emission direction, the unidirectional anisotropy. The generated THz time signal undergoes a phase shift of π when the sample is rotated from 0°  to 360°, and it returns to its original state upon completing a full rotation to 360°, as shown in Figure S2 (Supporting Information). These findings validate that the proposed THz emitter is indeed an exchange bias‐influenced THz.^[^ ^1^ ^]^

The thickness‐dependent (t_MnPt_) terahertz spintronics magnetometry (TSM) measurements (Figure S3, Supporting Information) reveal the variation of both exchange bias and the coercive field with different MnPt thicknesses. The exchange bias field is defined as H_ex_ = (H_R_ + H_L_)/2, and the coercivity as H_c_ = (H_R_−H_L_)/2, where H_R_ and H_L_ correspond to the fields at which the forward and backward branches of the hysteresis loop intersect the field axis.^[^ ^24^, ^25^ ^]^ The thickness of the CoFeB layer is set to 3 nm for the following three critical reasons. First, a thicker FM layer may diminish interfacial exchange interactions, reducing the exchange field and coercivity.^[^ ^26^, ^27^ ^]^ Second, a thicker FM layer can expedite spin‐current attenuation due to spin relaxation and scattering within the bulk of the FM material, preventing the spin current from reaching the AFM layer.^[^ ^28^ ^]^ Finally, absorption of the THz signal from a thicker FM layer will reduce the emission efficiency.^[^ ^29^ ^]^ Meanwhile, the thickness of the AFM layer plays a key role in manipulating the exchange bias‐induced THz emission. As the AFM layer thickness increases, the exchange bias field increases gradually due to enhanced pinning of the FM spins along the anisotropy axis of the AFM because of the FM and AFM exchange interaction at the interface.^[^ ^26^ ^]^ This enhanced exchange bias lowers the energy barrier for domain reversal in the FM layer, leading to a gradual reduction in coercivity,^[^ ^4^, ^26^ ^]^ which agrees with our results in Figure 1e.

**Figure 2:**
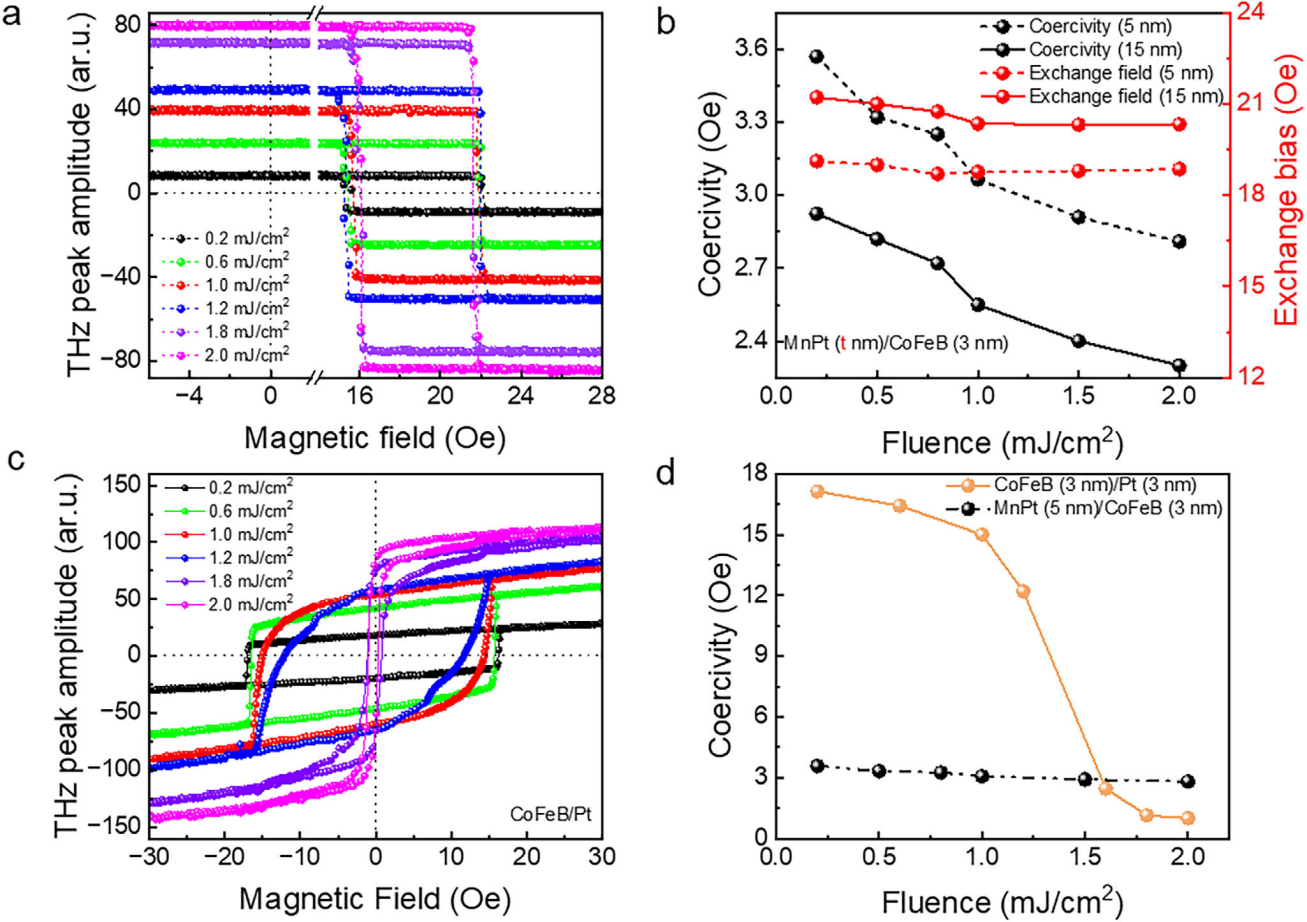
Robustness of magnetization dynamics in an EBC‐STE with laser fluence. a) Laser‐fluence‐dependent THz‐H hysteresis of the MnPt (5 nm)/CoFeB (3 nm) sample shows a reduced hysteresis width with increasing pump fluence, while the center position remains unchanged, indicating that the exchange bias is unaffected. b) Fluence‐dependent coercivity and exchange bias for MnPt (t nm)/CoFeB (3 nm) with t = 5 nm and 15 nm reveal localized decoupling of magnetization. c) For comparison, a conventional STE of CoFeB (3 nm)/Pt (3 nm) is studied to examine the resilience of magnetization under external laser fluence. The THz‐H hysteresis signifies the faster shrinking of the hysteresis width with the laser fluence, and the corresponding d) coercive field variation of the conventional STE and the exchange bias coupled STE (MnPt (5 nm)/CoFeB (3 nm) as a function of pump fluence indicates that exchange bias STE retains magnetization more efficiently than laser perturbation compared to the conventional STE.

To uncover the robustness of magnetic behavior and interfacial stability, we perform fluence‐dependent TSM measurements on EBC‐STE and a conventional FM/HM STE. For the EBC‐STEs, the fluence‐dependent TSM measurements show nearly rectangular terahertz‐H hysteresis (THz‐H) loops, as shown in **Figure** **2**a. These results indicate two significant consequences. First, an increase in spin current (**j**_s_) generation with pump fluences and their diffusion from the FM layer into the AFM layer, resulting in a linear increase in the THz peak amplitude with fluence as shown in the Figure S4 (Supporting Information), and second, a shrinking in the width of the THz‐H hysteresis loops, attributed to the decrease in the coercive field of the FM layer, while the exchange field remains nearly constant. The quantitative variation of the coercivity and the exchange field for t_MnPt_ = 5 and 15 nm with pump fluences is shown in Figure 2b.

For comparison, we also investigate a conventional CoFeB (3 nm)/Pt (3 nm) STE sample, as shown in Figure 2c. The change in the coercive fields in both samples is shown in Figure 2d. In this case, the rate of change of the coercive field per mJ/cm^2^ of pump fluence is much lower in the EBC‐STE than in the conventional STE. This minor reduction in coercivity in EBC‐STE with increasing laser fluence may arise from the lower magnetic energy loss due to thermal heating and the pinning of FM moments, which mitigates the impact of the thermal effect.^[^ ^30^ ^]^ In contrast, conventional CoFeB/Pt heterostructures exhibit a sharp reduction in coercivity at fluences ≥ 1.2 mJ/cm^2^. Ultrafast laser heating induces nonlinear thermal expansion or contraction that transiently modifies the magnetic properties of the FM layer and its interface with the heavy metal.^[^ ^6^, ^31^ ^]^ As the fluence crosses this threshold, the transient temperature at the interface may approach critical values such as the Curie temperature of the FM layer, drastically reducing magnetic anisotropy and weakening domain walls.^[^ ^30^, ^31^ ^]^ At higher fluence, these nonlinear thermal and magnetoelastic effects dominate, leading to distorted hysteresis loops and a pronounced increase in THz emission amplitude due to enhanced spin current generation, while the coercivity falls sharply.

**Figure 3:**
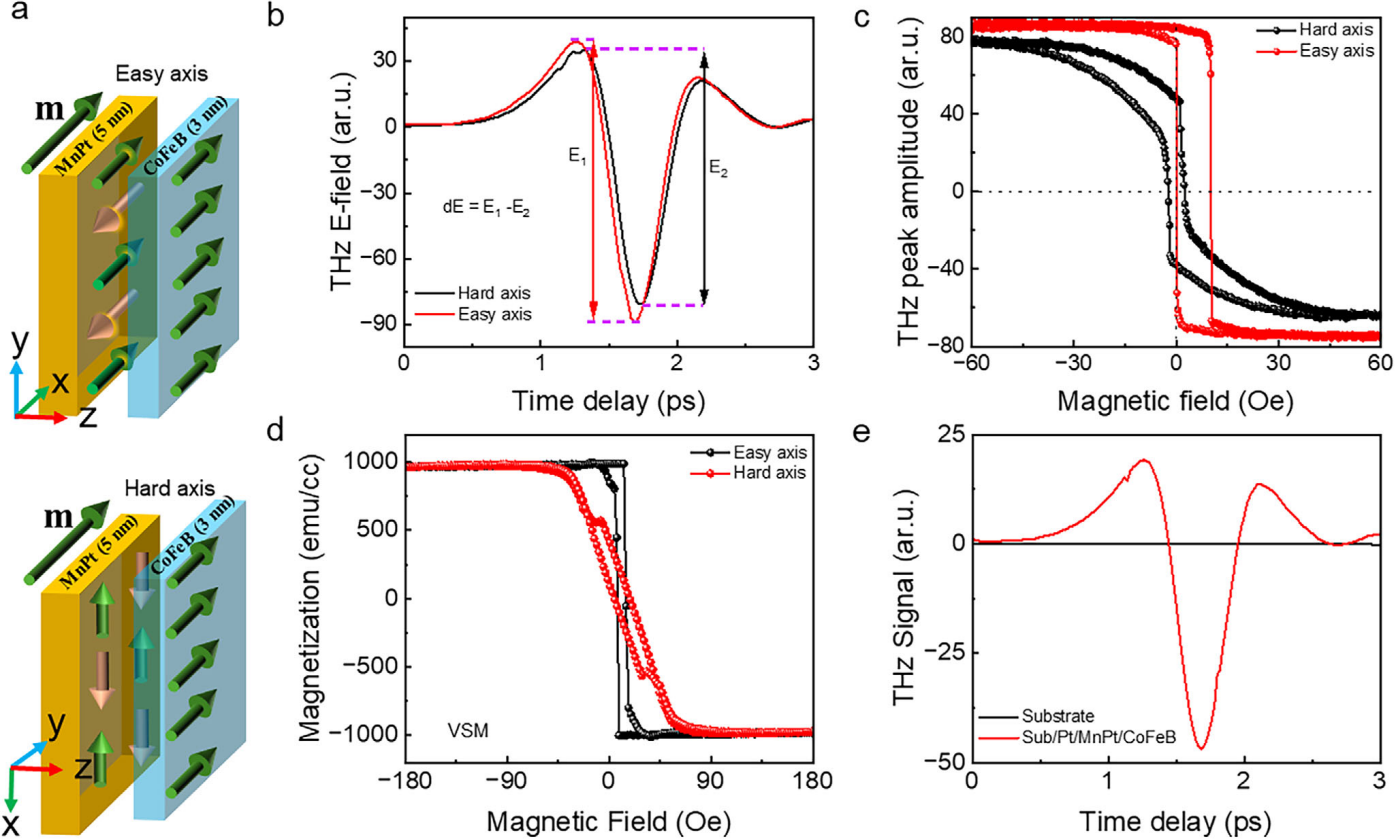
Active contribution of MnPt to THz emission. a) The pictorial representation of FM and AFM spin configurations shows the easy and hard axis directions of the EBC‐STE under a fixed saturation magnetic field of the FM material. b) The THz time‐domain signal is measured along the easy and hard axis directions of the sample, revealing a significant difference in peak‐to‐peak amplitude (dE = E_1_–E_2_), where E_1_ and E_2_ represent the THz peak‐to‐peak amplitudes of the emitted THz signals along the easy and hard axes, respectively. c) The TSM measurements capture this amplitude difference, whereas d) the VSM measurements provide identical saturation magnetization along both axes. Since TSM probes ultrafast dynamics, contributions from MnPt or the substrate are directly involved in the THz emission. In contrast, VSM is a static measurement that excludes ultrafast transient magnetization, thereby confirming equal saturation magnetization of CoFeB along both directions. This rules out unequal CoFeB magnetization as the source of the difference and indicates contributions from other layers. e) The substrate contribution is negligible compared to the EBC‐STE signal, signifying that the additional contribution originates from the MnPt layer.

We design a novel experiment with two different sample configurations under an external magnetic field. In the first configuration, the sample is subjected to an external magnetic field along the easy axis direction of the EBC‐STE, with the applied field (H_applied_) greater than the saturation field (H_sat_) of the CoFeB layer. In the second configuration, the sample is rotated by 90°, aligning it along its hard axis relative to the magnetization direction. The corresponding spin configurations of the MnPt and CoFeB layers are schematically shown in the top and bottom panels of **Figure** **3**a, respectively. The THz time domain signals corresponding to these two different configurations are shown in Figure 3b. In these measurements of THz time domain signals, we observe a significant difference in the THz peak‐to‐peak amplitudes (P─P Amp.) (dE = E_1_–E_2_) between the two configurations (easy‐axis vs hard‐axis alignment). In the absence of an external magnetic field, rotating the sample typically reorients the FM magnetization along the easy direction of the EBC‐STE. This rotation of FM spins reduces the THz peak amplitude gradually and causes its phase to flip after a full 180  azimuthal rotation. This phase flipping happens because the exchange interaction at the AFM/FM interface pins the FM spins, forcing them to rotate with the sample. In contrast, when an external magnetic field stronger than the saturation field (H_applied_ > H_sat_) is applied, the FM magnetization remains fixed along the direction of the magnetic field. If the THz emission originates solely from spin‐polarized hot electrons generated in the FM layer, the THz signal amplitude would be the same for both configurations (dE≈0). This difference in THz signal amplitude indicates an additional contribution to the THz generation mechanism beyond the ISHE, which accounts for the observed amplitude difference. The TSM measurements (Figure 3c) also evident the unequal saturation amplitude along both axes. In contrast, static magnetometry using a Vibrating Sample Magnetometer (VSM) shows identical saturation magnetization along both axes (Figure 3d). Since TSM captures ultrafast dynamics, contributions from MnPt or the substrate are directly involved in the THz emission. VSM, on the other hand, reflects the static magnetization of CoFeB and confirms that it is identical along both directions. This excludes unequal CoFeB magnetization as the origin of the THz amplitude difference and points instead to contributions from other layers. To confirm whether this THz amplitude difference comes from the substrate, we measured the substrate as shown in Figure 3e, which does not show a detectable signal in comparison to the EBC‐STE sample. These results exclude the substrate contribution, leaving the MnPt layer as the most likely source of the observed amplitude difference. However, MnPt does not carry a net magnetic moment in its static state, raising the important question of how it contributes to THz emission. This points to an unconventional emission mechanism and may come from the ultrafast tilting of the Néel vector driven by the laser‐induced optical torque.

**Figure 4:**
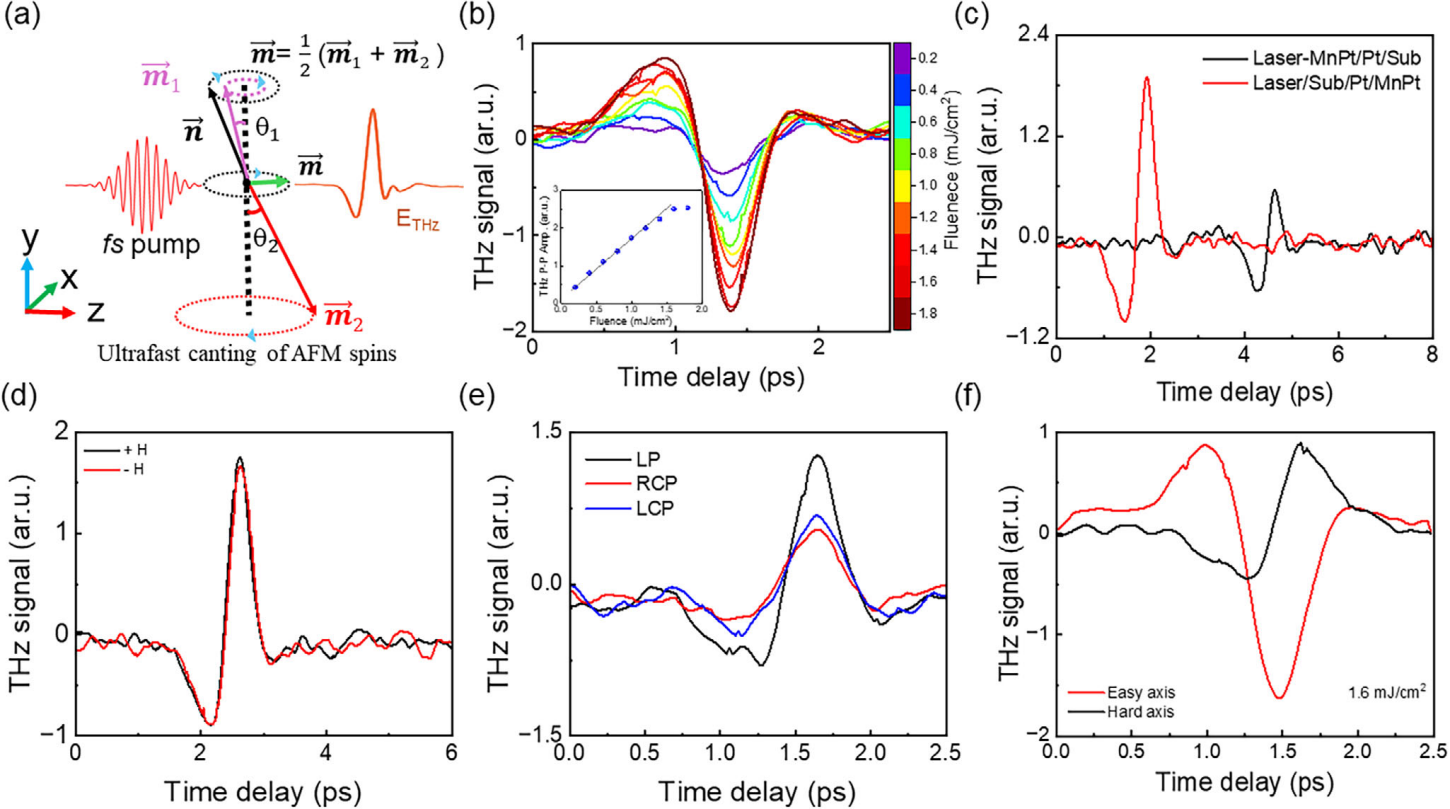
The ultrafast light‐driven canting of the Neel vector in Pt/MnPt. a) Schematic representation of the ultrafast light‐induced transient magnetization dynamics in the antiferromagnet, arising from canting of the antiferromagnetic spin sublattices. Here, m and n denote the net magnetic moment and Néel vector, respectively; their direction and magnitude depend on the canting angles (θ_1_,θ_2_) of the sublattice magnetic moments. b) The THz time‐domain signal from a Pt (3 nm)/MnPt (5 nm) sample, with the inset displaying that the emitted THz signal scales linearly with pump fluence. c) Comparison of THz emission when excited from the film side vs the substrate side. The absence of phase reversal suggests the source is not the inverse spin Hall effect. The delay and amplitude reduction when exciting from the film side are due to signal propagation through the Pt and substrate, supporting the canting‐induced transient magnetization as the emission mechanism. d) The THz signal is insensitive to low external magnetic fields (H_ext_ > H_Sat, CoFeB_), indicating the emission arises from transient magnetization dynamics that preserve the antiferromagnetic properties due to strong anisotropy and exchange interaction. e) The emitted THz signal strongly depends on the pump polarization direction, confirming that the emission originates from antiferromagnetic spin dynamics. f) The opposite phases of THz signals measured along two orthogonal azimuthal directions in the Pt/MnPt sample explain the origin of the THz P─P amplitude difference (dE) observed in the exchange bias sample. This confirms that the MnPt layer actively contributes to the THz emission under ultrafast excitation.

We designed a complementary experiment using a Pt/MnPt sample without the CoFeB layer to investigate how a collinear AFM with zero net magnetization can emit THz radiation. The distinct THz signal detected from the Pt/MnPt sample (**Figure** **4**b) confirms ultrafast manipulation of the Néel vector and demonstrates the intrinsic role of the AFM layer in THz emission. The mechanism of THz emission in MnPt is further supported by a pump‐direction‐dependent study. The MnPt layer exhibits in‐phase THz signals whether the sample is excited from the MnPt side or the substrate side, as shown in Figure 4c, which excludes the inverse spin Hall effect (ISHE) as the origin of the emission. Additionally, the observed time delay and reduced amplitude of the THz signal for front‐side excitation (Figure 4c) reflect propagation effects through the Pt layer and substrate. These findings confirm that Pt acts only as a buffer layer and that no spin‐to‐charge conversion via ISHE occurs.

Building on this observation, we propose a theoretical framework to provide deeper insight into the ultrafast magnetization dynamics in MnPt. The THz emission arises from laser‐fluence‐induced canting of the sublattice spin order, specifically a tilting of the Néel vector ***n*** from its equilibrium orientation (Figure 4a). The Néel vector ***n** =* (***m**_1_–**m**_2_*)/2^[^ ^32^ ^]^ represents the order parameter that describes the alignment of the two antiparallel sublattice magnetizations, ***m**_1_* and ***m**_2_*. Under equilibrium, ***n*** is fixed by magnetic anisotropy and exchange interactions. When an ultrafast optical pulse interacts with the MnPt, it can induce a laser optical torque (LOT) due to the strong spin‐orbit coupling present in this material. These torques act oppositely on the two sublattices, causing the spins to cant away from their equilibrium antiparallel alignment as shown in Figure 4a. The ultrafast laser pulse generates this torque via a staggered opto‐magnetic field arising from second‐order electric field coupling. The LOT can be expressed as^[^ ^34^, ^35^ ^]^

(1) $$\tau_{LOT}\propto {\left|E\right|}^{2}\chi_{ijklm}\in_{j}\in_{k}^{*}n_{l}n_{m}$$

where *ϵ_j_* is the *j*th Cartesian component of the electric field and *n_j_* is the *j*th Cartesian component of the Néel vector, and *χ_ijklm_* are the corresponding susceptibility components. The laser‐induced torque is linearly proportional to the laser fluence. The canting dynamics of the Néel vector are governed by coupled Landau‐Lifshitz‐Gilbert (LLG) equations for the sublattice magnetizations. For the Néel vector, under the influence of the ultrafast torque, the equation of motion is^[^ ^36^ ^]^

(2) $$\frac{dn}{\mathrm{dt}}=-\gamma n\times H_{\mathrm{eff}}+\frac{\alpha }{M_{s}}n\times \frac{dn}{\mathrm{dt}}+\tau_{\mathrm{LOT}}$$

where *γ* is the gyromagnetic ratio, ***H**_eff_* is the effective magnetic field, *α* is the Gilbert damping parameter, and *M_s_* is the sublattice saturation magnetization. Due to the canting, a transient magnetization emerges, given by $M\propto n\times \frac{dn}{\mathrm{dt}}$.^[^ ^37^ ^]^ The canting‐induced transient magnetic moment in MnPt produces a transient current that acts as the source of the emitted THz radiation. At low fluence, the laser‐induced optical torque is proportional to the laser fluence, generating a transient magnetization and THz signal that scales linearly with fluence.^[^ ^34^ ^]^ However, at high fluence, the maximum canting angle allowed by the strong exchange interaction and thermalization effects limits the reorientation of the Néel vector, resulting in the saturation of the THz peak‐to‐peak amplitude.

In further interpreting the transient magnetization dynamics in MnPt, we have performed systematic magnetic field and pump polarization‐dependent studies. Antiferromagnets typically exhibit insensitivity to low external magnetic fields due to their strong internal exchange coupling and high magnetic anisotropy energy.^[^ ^10^ ^]^ Consistent with this, the emitted THz signal from MnPt is found to be independent of applied magnetic fields up to ±200 Oe, as shown in Figure 4d. This observation aligns with the substantial magnetic anisotropy energy of MnPt, which prevents such low fields from altering their magnetic state. Furthermore, the THz emission exhibits a pronounced dependence on the pump polarization. As seen in Figure 4e, circularly polarized excitation results in significantly reduced THz emission compared to linearly polarized excitation. This behavior supports the mechanism in which the laser‐induced optical torque (LOT) induces a canting of the Néel vector in collinear AFM MnPt, thereby generating a transient magnetic moment that emits the observed THz radiation. The strong polarization dependence rules out purely thermal effects and confirms the ultrafast, coherent control of the Néel vector by the optical pump.

However, THz emission from MnPt does not yet explain the cause of P─P Amp. difference (dE) observed in Figure 3b. The azimuthal dependence study shows that the phase of the THz signal reverses with a 90°  sample rotation, and the amplitude is stronger in one direction than in the rotated configuration (Figure 4f). This opposite‐phase behavior in two perpendicular azimuthal directions explains the amplitude difference observed in the EBC‐STE sample. Considering this AFM contribution, the emitted signals from the EBC‐STE sample along the easy and hard axes can be expressed by

(3) $$E_{Easy}\propto E_{FM}+E_{AFM,Easy}$$

(4) $$E_{Hard}\propto E_{FM}-E_{AFM,Hard}$$

where, *E_FM_* *and* *E*_*AFM*,*Easy*/*Hard*_ denote the THz electric field contribution of the FM and AFM layers, respectively. These expressions elucidate that the dE observed in the EBC‐STE mainly originates from the contribution of the AFM layer. Thus, we successfully reveal the origin of the amplitude difference dE in our exchange bias THz emitter by accounting for the antiferromagnetic contribution to the THz emission.

In summary, we propose and experimentally demonstrate the ultrafast magnetization dynamics of MnPt within an exchange‐biased AFM‐FM heterostructure. Our results provide direct evidence of a transient magnetic moment in MnPt, originating from laser‐induced optical torque that cants the Néel vector and generates a detectable nonequilibrium magnetization. Besides the uncovering of the fundamental physics of ultrafast Neel vector control, we demonstrate the unique characteristics of exchange bias through TSM and magnetic field‐dependent studies. The exchange‐bias coupling remains remarkably robust under ultrafast optical excitation, endowing this emitter with superior stability compared to conventional spintronic emitters. This work opens new paths for designing active, high‐speed terahertz spintronic devices based on ultrafast optical control of antiferromagnetic order.

## 3 Experimental Section

### THz Emission Spectroscopy Measurement

In our THz emission spectroscopy experiments, a Ti: sapphire femtosecond laser source was used to optically pump the THz emitter. This laser operates at a repetition rate of 1 kHz, delivering pulses with a duration of 45 fs, centered at a wavelength of 800 nm, and a maximum output power of 4.3 W. The laser beam was split into two paths: a pump pulse and a probe pulse. The pump pulse excites the EBC‐STE (exchange‐bias‐coupled spintronic terahertz emitter) sample, while the probe pulse was used to detect the emitted THz radiation. Detection was achieved through electro‐optic sampling, employing a 1 mm ZnTe crystal oriented along the <110> axis as the detector. To investigate the THz hysteresis behavior of the sample, an external in‐plane magnetic field was applied, which was generated under the application of an electric voltage. The THz peak amplitude was measured across a range of magnetic field strengths, varying from positive to negative polar directions, allowing us to observe the response of the sample under different laser fluences. The detailed schematic representation of the experimental setup is shown in Figure S1 (Supporting Information).

### Sample Fabrication

The exchange bias‐coupled MnPt/CoFeB heterostructure was fabricated in a Singulus Timaris magnetic sputtering system maintained at a base pressure of < 1 × 10^−8^ mbar. To ensure the antiferromagnetic (AFM) spin ordering in the MnPt layer, a 3 nm Pt seed layer was first deposited on the substrate. This seed layer promotes the crystalline quality and facilitates the proper spin configuration of the subsequent MnPt layer. Following this, the AFM MnPt and FM CoFeB layers were sequentially deposited. The deposition process was carried out at room temperature. The CoFeB layer was deposited with an applied in‐plane bias field of 120 Oe along a desired direction. A 3 nm thick SiO_2_ capping layer was deposited to prevent oxidation.

## Conflict of Interest

The authors declare no conflict of interest.

## Author Contributions

S.J., S.S.M., and R.S. conceived the idea. S.J. and R.S. designed the experiments with input from S.S.M. S.J. performed all the THz emission measurements and analyses with the help of S.S.M. J.L. designed the samples. S.J. and R.S. wrote the manuscript after input from all authors. All authors approved the publication of the final version of the manuscript. R.S. supervised the overall project.

## Supporting information



Supporting Information

## Data Availability

The data that support the findings of this study are available from the corresponding author upon reasonable request.
