# Supplementary material for: Ultrafast Control of Néel Vector in Collinear Antiferromagnet MnPt
Source: Adv Sci (Weinh). 2025 Nov 10;13(4):e19395. doi: 10.1002/advs.202519395 (PMC12822418; doi:10.1002/advs.202519395)
Supplement: Supplementary file 1 — Supporting Information [file ADVS-13-e19395-s001.docx]

**Supplementary Information**

**Ultrafast Control of Néel Vector in Collinear Antiferromagnet MnPt**

*Sambhu Jana^1,2^, Sobhan Subhra Mishra^1,2^, James Lourembam^3^,* *and Ranjan Singh^4^**

^1^ Division of Physics and Applied Physics, School of Physical and Mathematical Sciences, Nanyang Technological University, Singapore, 637371, Singapore

^2^ Centre for Disruptive Photonic Technologies, The Photonics Institute, Nanyang Technological University, Singapore, 639798, Singapore

^3^ Institute of Materials Research and Engineering (IMRE), Agency for Science, Technology and Research (A*STAR), 2 Fusionopolis Way, Singapore, 138364, Singapore

^4^ Department of Electrical Engineering, University of Notre Dame, Notre Dame, IN 46556, USA

* Corresponding author: [rsingh3@nd.edu](mailto:rsingh3@nd.edu)

**SI-1: Experimental set-up:**


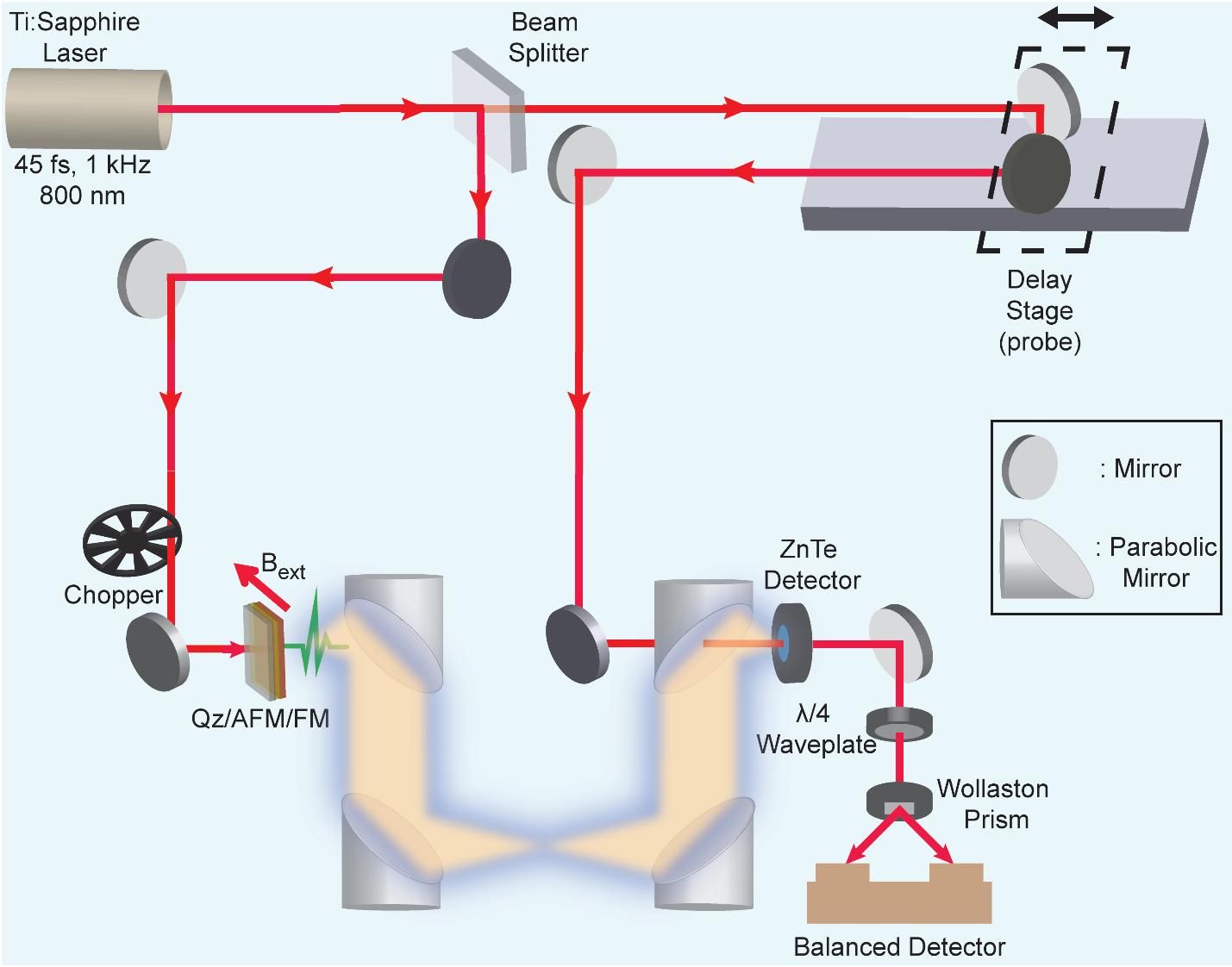


**Figure S1.** **Schematic of the terahertz emission spectroscopy setup.** Typically, a 45-fs pulse width and 1 kHz repetition rate femtosecond laser source is used to excite EBC-STE. The central wavelength is 800 nm. The ultrafast laser pulse is directed onto a sample, exciting charge carriers and spin dynamics that generate broadband THz radiation. Optical delay lines control the pump-probe timing, allowing time-resolved measurements. The emitted THz waves are collected and focused using parabolic mirrors and then detected using a 1mm thick ZnTe nonlinear crystal through electro-optic sampling. A lock-in detection scheme enhances the signal-to-noise ratio, ensuring precise spectral analysis. The system is often housed in a controlled environment to minimize external noise and optimize measurement accuracy.

**SI-2: Azimuthal dependence on THz emission in an EBC-STE:**

The unidirectional anisotropy in THz emission from exchange bias systems arises from the interfacial exchange coupling between an AFM and a FM^1^. When an FM is interfaced with an AFM, the exchange interaction at the interface pins the FM magnetization along a preferred direction, breaking the in-plane rotational symmetry and establishing a unidirectional anisotropy, the signature of exchange bias^2,3^. Upon femtosecond laser excitation, ultrafast spin currents are generated in the FM and injected into the adjacent heavy metal (HM) layer, where they are converted into charge currents via the ISHE, resulting in THz emission. The direction and polarity of the emitted THz field are directly determined by the direction of the pinned magnetization set by the exchange bias. This means that, unlike in unbiased systems where the emission is symmetric with respect to magnetization reversal, the exchange bias introduces a preferred emission direction, the unidirectional anisotropy.





**Figure S2.** **Azimutation variation.** The azimuthal dependence of the THz emission in the absence of an external magnetic field. The generated THz time signal undergoes a phase shift of π when the sample is rotated from 0^°^ to 180^°^, and it returns to its original state upon completing a full rotation to 360^°^.

**SI-3: Thickness-dependent TSM:**

Terahertz spintronic magnetometry (TSM)^4^ is an ultrafast, contact-free technique that utilizes the emission of terahertz (THz) radiation from spintronic heterostructures to probe and characterize the in-plane magnetization dynamics of ferromagnetic thin films. The amplitude of the emitted THz pulse is directly proportional to the magnetization of the sample, which allows the construction of a THz-H hysteresis loop by sweeping the external magnetic field and recording the THz pulse amplitude as a function of field strength. This THz-H hysteresis closely mirrors the conventional magnetization (M-H) hysteresis measured by vibrating sample magnetometry (VSM)^5^, but with the added advantages of ultrafast temporal resolution and the ability to probe dynamic magnetic phases without electrical contacts or substrate background corrections. As we increase the thickness of the MnPt layer, the hysteresis loop shifts more towards the field axis, increasing the exchange energy as described in Fig. 1e in the main manuscript.


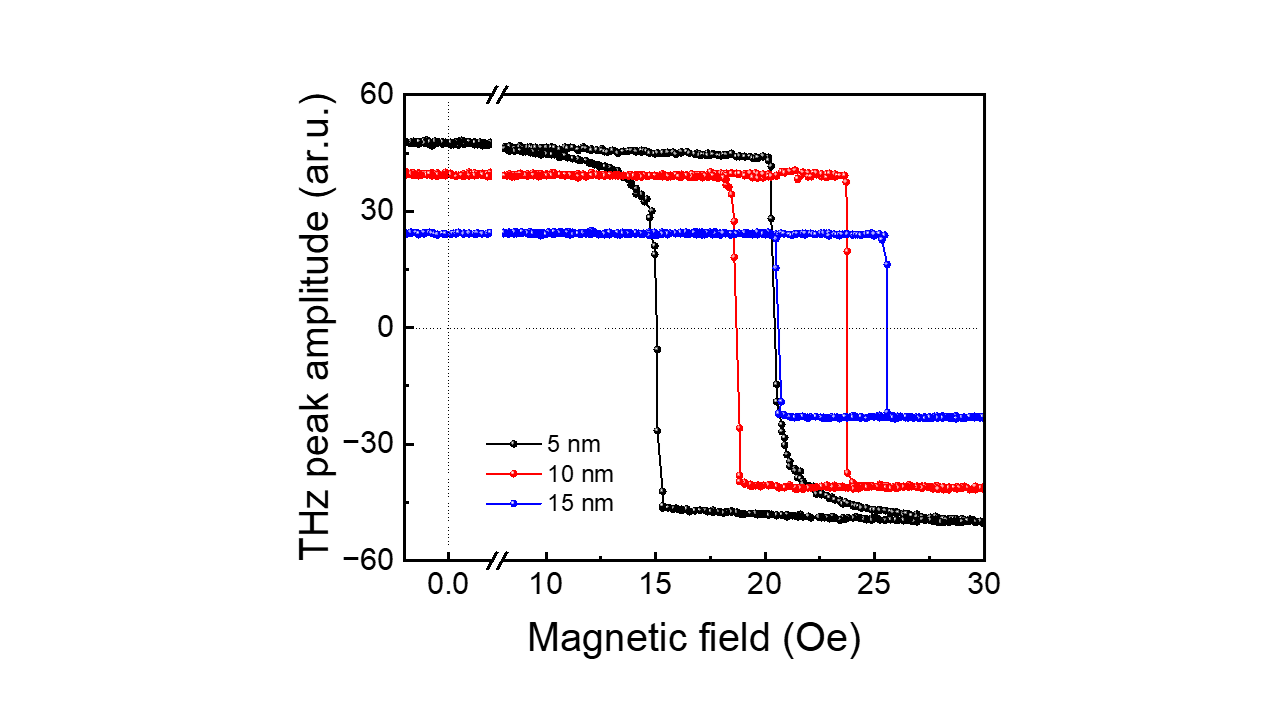


**Figure S3.** **Thickness-dependent THz-H hysteresis.** The results indicate a shrinking of the hysteresis curve with increasing MnPt thickness, accompanied by an enhanced hysteresis shift due to the stronger exchange interaction at the interface.

**SI-4: Role of fluence and thickness of MnPt on THz emission:**





**Figure S4.** **Role of thickness and fluence on THz emission.** The terahertz peak-to-peak amplitude as a function of pump fluence is analyzed for different MnPt thicknesses, including 5 nm, 10 nm, and 15 nm. The THz peak amplitude increases linearly with the laser pump fluence. The thickness variation shows that a thicker AFM layer can enhance spin relaxation, reducing emitted THz intensity, whereas an optimized AFM thickness can maximize spin conversion efficiency and THz emission.

The linear increase of THz emission with pump fluence in a Pt/MnPt/CoFeB heterostructure arises because, at low to moderate fluence, the number of photoexcited carriers and the efficiency of ultrafast spin current generation both scale proportionally with the absorbed laser energy.^6^ In such spintronic heterostructures, femtosecond laser pulses excite the CoFeB layer, generating a spin-polarized current that is either converted to a charge current via the inverse spin Hall effect in the adjacent MnPt layer. Experimental studies confirm that the THz emission amplitude shows a linear dependence on pump fluence up to a certain threshold, reflecting the direct relationship between the excitation density and the resulting spin/charge current responsible for THz generation. This linear regime persists until other effects, such as saturation of available states, heating, or non-linear absorption, become significant, but in the initial range, the process is governed by the proportional increase in excitation and subsequent THz emission.^7,8^ In addition, the amplitude of the emitted THz signal decreases gradually as we increase the thickness of the MnPt layer due to absorption of the THz signal by the metallic layer.

**References:**

1. Radu, F. & Zabel, H. Exchange Bias Effect of Ferro-/Antiferromagnetic Heterostructures. in *Springer Tracts in Modern Physics* vol. 227 97–184 (2008).

2. Leighton, C. *et al.* Thickness-dependent coercive mechanisms in exchange-biased bilayers. *Phys. Rev. B - Condens. Matter Mater. Phys.* **65**, 644031–644037 (2002).

3. Stamps, R. L. Mechanisms for exchange bias. *J. Phys. D. Appl. Phys.* **33**, R247–R268 (2000).

4. Agarwal, P. *et al.* Terahertz spintronic magnetometer (TSM). *Appl. Phys. Lett.* **120**, (2022).

5. Zhang, W. *et al.* Ultrafast terahertz magnetometry. *Nat. Commun.* **11**, 4247 (2020).

6. Seifert, T. *et al.* Efficient metallic spintronic emitters of ultrabroadband terahertz radiation. *Nat. Photonics* **10**, 483–488 (2016).

7. Koleják, P. *et al.* Maximizing the Electromagnetic Efficiency of Spintronic Terahertz Emitters. *Adv. Photonics Res.* (2024) doi:10.1002/adpr.202400064.

8. Agarwal, P. *et al.* Secondary Spin Current Driven Efficient THz Spintronic Emitters. *Adv. Opt. Mater.* **11**, (2023).
